# A dynamical systems approach for estimating phase interactions between rhythms of different frequencies from experimental data

**DOI:** 10.1371/journal.pcbi.1005928

**Published:** 2018-01-16

**Authors:** Takayuki Onojima, Takahiro Goto, Hiroaki Mizuhara, Toshio Aoyagi

**Affiliations:** Graduate School of Informatics, Kyoto University, Kyoto, Japan; University of Pittsburgh, UNITED STATES

## Abstract

Synchronization of neural oscillations as a mechanism of brain function is attracting increasing attention. Neural oscillation is a rhythmic neural activity that can be easily observed by noninvasive electroencephalography (EEG). Neural oscillations show the same frequency and cross-frequency synchronization for various cognitive and perceptual functions. However, it is unclear how this neural synchronization is achieved by a dynamical system. If neural oscillations are weakly coupled oscillators, the dynamics of neural synchronization can be described theoretically using a phase oscillator model. We propose an estimation method to identify the phase oscillator model from real data of cross-frequency synchronized activities. The proposed method can estimate the coupling function governing the properties of synchronization. Furthermore, we examine the reliability of the proposed method using time-series data obtained from numerical simulation and an electronic circuit experiment, and show that our method can estimate the coupling function correctly. Finally, we estimate the coupling function between EEG oscillation and the speech sound envelope, and discuss the validity of these results.

## Author summary

In this paper, we propose an estimation method to identify a dynamical system from rhythmic time-series data. Rhythmic activities have been observed frequently and are synchronized in various fields, and synchronization is an important topic in nonlinear science. It is well known that such synchronization can be described theoretically by a phase oscillator model under the condition that the rhythmic activities can be considered weakly coupled limit-cycle oscillators. Based on this theory, we propose a method to identify the interaction between rhythmic activities as a network of phase oscillators. A practical advantage of the proposed method is that, without detailed modeling, we can extract the phase oscillator model directly from time-series data. For the above theoretical and practical reasons, this method can be applied to rhythmic data from a wide range of fields. In this study, we have focused on human brain activities in which electroencephalography (EEG) signals are often synchronized with each other and with external periodic stimuli. We demonstrate that the proposed method can successfully estimate the interaction between EEG activity and speech rhythm. Consequently, the proposed method can reveal the role of neural synchronization.

## Introduction

Synchronization of neural oscillations is considered an important activity that can help reveal the mechanisms underlying various cognitive functions. Neural oscillation is a rhythmic neural activity and is usually observed by electroencephalography (EEG). Neural oscillations are classified into a few frequency bands (e.g. delta, theta and alpha frequency bands) and are synchronized within the same-frequency band between different brain areas during various cognitive tasks [1–4]. Synchronization of oscillations of the same frequency is considered to integrate distributed brain activities [5] and regulate communication between distant neural groups [6, 7].

Synchronization between slow and fast oscillations (cross-frequency synchronization) also appears during a few cognitive tasks [8–11]. In particular, 1:*p* phase synchronizations (*p* is an integer) can be observed in the resting state, mental arithmetic tasks, and working memory tasks [12–17], and may integrate activities over different time scales [18]. 1:*p* phase synchronization refers to phase locking of a single cycle of one oscillation to *p* cycles of the other oscillation. Although 1:*p* phase synchronization is considered important from the perspective of brain function, to the best of our knowledge, there is no effective and practical method to analyze the 1:*p* phase synchronization mechanism.

Various methods to identify this synchronization have been used in EEG studies. For example, the phase locking index is used frequently to identify phase synchronization. This index measures the temporal consistency or intertrial variability of the phase difference between different brain areas or cross-frequency oscillations [2, 19–22]. In addition, the directional connectivity between neural oscillations has been evaluated in terms of transfer entropy [23, 24]. Transfer entropy evaluates the directed transfer of information between two random processes. Many previous studies have examined the roles of neural oscillation using these methods. However, these methods could not reveal how neural synchronization is achieved by a dynamical system. Therefore, we have developed a method to identify a dynamical system for synchronization.

It is widely believed that the dynamical system of EEG activity can be described by the neurophysiological model of a cortical column [25, 26]. If this dynamical system can be explained by a weakly coupled oscillator, the corresponding neurophysiological model can be described using the phase oscillator model in which each oscillator is described by only one variable, i.e., the phase [27]. Some previous studies have provided estimation methods to derive the phase oscillator model directly from time-series data without detailed modeling [28–34]. However, such methods cannot be applied to cross-frequency synchronization data. Therefore, we extend previous methods to make them applicable to 1:*p* phase synchronization.

In this paper, we describe an extended method to explain 1:*p* phase synchronization based on the phase oscillator model and verify the reliability of the estimation method through numerical simulation and an electronic circuit experiment. Then, we apply the proposed method to EEG oscillation and speech sound. Speech rhythms are synchronized with neural activity in a listener’s brain [35], and speech rhythm consists of a few important linguistic components (e.g., syllable and prosody). It is believed that synchronization between neural oscillation and linguistic rhythm contributes to parsing continuous speech [36] and predicting the timing of linguistic component production [35] [37]. Furthermore, the causality between EEG activity and speech sound is clear, whereas the causality among neural activities is generally unknown in advance. Therefore, we estimate interactions between EEG activity and speech sound to confirm the validity of the estimation results and demonstrate that the proposed method can successfully estimate the dynamical system based on EEG data.

## Materials and methods

### Ethics statement

The scalp EEG experiment was approved by the ethics committee of the Unit of the Integrated Studies of the Human Mind, Kyoto University (24-p-19). Participants provided written informed consent according to the Declaration of Helsinki and were paid for their participation.

### Estimation of phase coupling functions for cross-frequency synchronization

Neural oscillations can be observed easily from EEG data, and many EEG studies have reported various types of synchronization [8, 38], which can be roughly divided into same- and cross-frequency synchronization. A few experimental results suggest that same-frequency phase-phase synchronization plays a role in modulating neuronal interaction [6, 7]. In contrast, cross-frequency synchronization is considered to play a role in the integration of activities over different time scales [18]. However, it is unclear how these synchronizations, particularly 1:*p* phase synchronization, are achieved by a dynamical system. Therefore, we developed an effective method to identify the dynamical system that performs these synchronizations.

In general, synchronization of neural oscillation is thought to be described by a network of limit-cycle oscillators, which can be described generally by the multidimensional differential equation $\frac{dX_{i}}{dt}=F_{i}(X_{i})+\sum_{j\neq i}^{N}G_{ij}(X_{i},X_{j})$, where ***X***_*i*_ denotes the multidimensional state of the *i*-th oscillator, such as membrane voltages and gate variables of ionic channel. We assume that a system ***X***_*i*_ can generate a limit-cycle oscillation by itself without external interaction. An EEG signal is thought to be generated by some neuronal system consisting of many interacting neurons. In this context, it is plausible that the neuronal system of an EEG signal can be represented by the ***X***_*i*_ system. According to the phase reduction method, the limit-cycle oscillator can be characterized theoretically by a phase *ϕ* as a simple dynamical system with one degree of freedom. If the oscillators are weakly coupled, the dynamics of the networks among *N* oscillatory systems can be described by [27, 39]:

$$\frac{d\phi_{i}}{dt}=\omega_{i}+\sum_{j\neq i}^{N}\Gamma_{i,j}(\phi_{j}-\phi_{i}),$$ (1)

where *ω*_*i*_ is the natural frequency of the oscillator and Γ_*i*,*j*_ is a phase coupling function representing the influence from the *j*-th oscillator to the *i*-th oscillator. It is known theoretically that the phase coupling function depends only on the phase difference *ϕ*_*j*_−*ϕ*_*i*_. When the phase difference is constant over time, these oscillators are said to be synchronized. Specifically, the synchronization of same-frequency oscillators is referred to as 1:1 phase locking. Eq (1) can describe the 1:1 phase-locking state between rhythms in real systems.

Various synchronizations between slow and fast oscillators, e.g., theta (4–8 Hz) and gamma (>30 Hz) EEG activities, have been observed ubiquitously, and they appear to play an important role in brain function [8–11]. In fact, 1:*p* phase locking has been observed ubiquitously in human EEG experimental studies during the resting state, mental arithmetic tasks, and working memory tasks [12–15]. However, 1:*p* phase synchronizations cannot be described by the model expressed by Eq (1). Therefore, we consider the 1:*p* phase-locking state among slow and fast oscillators. If 1:*p* phase locking occurs, the value of the phase difference *ϕ*_2_−*pϕ*_1_ is constant over time, where *ϕ*_1_ and *ϕ*_2_ are slow and fast phases, respectively. Using the phase reduction theory, we found that 1:*p* phase locking can be described as follows [39–41]:

$$\omega_{1}:\omega_{2}≅1:p,$$ (2)

$$\frac{d\phi_{1}}{dt}=\omega_{1}+\Gamma_{1,2}(\phi_{2}-{p\phi }_{1}),$$ (3)

$$\frac{d\phi_{2}}{dt}=\omega_{2}+\Gamma_{2,1}({p\phi }_{1}-\phi_{2}).$$ (4)

Here, we explain a simple case of two coupled oscillators described by Eqs (2)–(4). Note that many real rhythmic systems generally consist of many oscillators. We assume that the ratio of the natural frequencies of the two oscillators is close to some integer *p*. Note that, in this situation, the coupling function Γ_1,2_ depends on only the phase difference *ϕ*_2_−*pϕ*_1_.

In our approach, to investigate the nature of interactions between neuronal rhythms, we directly estimate both the natural frequencies *ω*_*i*_ and the phase coupling functions Γ_*i*,*j*_ from experimental time-series data. In addition, considering unavoidable sources of uncertainty (e.g., observational error or additional unknown disturbance to the system), we introduce independent Gaussian white noise *η*_*i*_(*t*) into the phase oscillator model as follows:

$$\omega_{i}:\omega_{j}≅p_{i}:p_{j},$$ (5)

$$\frac{d\phi_{i}}{dt}=\omega_{i}+\sum_{j\neq i}^{N}\Gamma_{i,j}(p_{i}\phi_{j}-p_{j}\phi_{i})+\eta_{i}(t).$$ (6)

Here, we assume that the independent Gaussian white noise *η*_*i*_(*t*) satisfies ⟨*η*_*i*_(*t*)⟩ = 0,⟨*η*_*i*_(*t*)*η*_*j*_(*t*′)⟩ = 2*D*_*i*_*δ*_*ij*_*δ*(*t* − *t*′), where *δ*_*ij*_ and *δ*(*t*) are the Kronecker delta and the Dirac delta functions, respectively. *D*_*i*_ indicates the noise strength and *p*_*i*_*ϕ*_*j*_−*p*_*j*_*ϕ*_*i*_ denotes the phase difference, where the *p* values are integers. Note that this phase oscillator model can explain *p*_*i*_:*p*_*j*_ synchronization (e.g., 2:3 phase synchronization and 2:7 phase synchronization). We estimate the phase oscillator model (Eq (6)) using almost periodic time-series data. In the following, we employ a straightforward extended version of a previously proposed method [28] and explain the outline of our method.

First, we transformed the experimentally-recorded signal *s*(t) into the phase time-series *θ*(t) by computing the analytic signal as follows:

$$A(t)e^{i\theta_{i}(t)}=s_{i}(t)+is_{i}^{H}(t),$$ (7)

where $s_{i}^{H}(t)$ denotes the Hilbert transformation of the recorded signal *s*_*i*_(*t*) [42], and *θ*(t) is the phase of the analytic signal. However, the variable *θ* is generally different from the phase *ϕ* in Eq (1) because, according to phase reduction theory, *ϕ* evolves linearly over time without interaction and noise. It is therefore necessary to transform *θ* into *ϕ*, as follows [30, 31]:

$$\phi (\theta )=2\pi \int_{0}^{\theta }f(\theta \prime )d\theta \prime ,$$ (8)

where *f*(*θ*) denotes the probability density distribution of *θ*.

Second, Bayesian linear regression [43, 44] is applied to estimate the parameters of the phase oscillator model given by Eq (6). Because the coupling function is periodic, we consider Fourier series expansion of the coupling function as:

$$\Gamma_{ij}(\psi_{ij}(t_{\tau }))=a_{ij}^{\left(0\right)}+\sum_{m=1}^{M_{ij}}\left\{a_{\mathrm{ij}}^{\left(m\right)}\cos\left(m\psi_{ij}(t_{\tau })\right)+b_{ij}^{\left(m\right)}\sin\left({m\psi }_{ij}(t_{\tau })\right)\right\},$$ (9)

where *ψ*_*i*,*j*_(*t*_*τ*_) is the extended version of the phase difference *p*_*i*_*ϕ*_*j*_(*t*_*τ*_) − *p*_*j*_*ϕ*_*i*_(*t*_*τ*_) at time *t*_*τ*_. The times *t*_*τ*_ are discrete points, *t*_*τ*_ = *t*_1_ + (*τ* − 1)Δ*t* for *τ* = 1,2,⋯,*T*, and Δ*t* is the sampling interval. In this expansion, *M*_*ij*_ denotes the Fourier series order for each coupling function, and the parameters *M*_*ij*_ control the complexity of the coupling function. The parameters *M*_*ij*_ can be determined by a model selection method based on logarithmic evidence, as explained later.

Finally, the proposed method estimates the model as follows:

$$\frac{d\phi_{i}}{dt}(t_{\tau })={\hat{\omega }}_{i}+\sum_{j\neq i}^{N}\sum_{m=1}^{M_{ij}}\{a_{\mathrm{ij}}^{\left(m\right)}\cos\left(m\psi_{ij}(t_{\tau })\right)+b_{ij}^{\left(m\right)}\sin\left({m\psi }_{ij}(t_{\tau })\right)\}+\eta_{i}(t_{\tau }),$$ (10)

where ${\hat{\omega }}_{i}=\omega_{i}+\sum_{j\neq i}^{N}a_{ij}^{\left(0\right)}$. Thus, the unknown model parameters are ${\left\{{\hat{\omega }}_{i},\;a_{ij}^{\left(m\right)},b_{ij}^{\left(m\right)}\right\}}_{i}$ and *D*_*i*_. Here, ${\left\{{\hat{\omega }}_{i},\;a_{ij}^{\left(m\right)},b_{ij}^{\left(m\right)}\right\}}_{i}$ denotes $\left\{{\hat{\omega }}_{i},\;a_{ij}^{\left(m\right)},b_{ij}^{\left(m\right)}|j=1,2,\cdots ,N,m=1,2,\cdots ,M_{ij}\right\}$. The phase velocity $\frac{d\phi_{i}}{dt}$ is a dependent variable in a standard linear regression problem that is computed from phase time-series data as {(*ϕ*_*i*_(*t*_*τ*+1_) − *ϕ*_*i*_(*t*_*τ*_))/Δ*t*}. Furthermore, an independent variable is computed by the phase difference as {cos(*mψ*_*ij*_(*t*_*τ*_)),sin(*mψ*_*ij*_(*t*_*τ*_))}_*i*_. Here, *η*_*i*_ is an independent and identically distributed random variable. This linear regression problem corresponds to maximizing the following likelihood function:

$$L\left(\{\phi_{i}(t_{\tau })\}|{\left\{{\hat{\omega }}_{i},a_{ij}^{\left(m\right)},b_{ij}^{\left(m\right)}\right\}}_{i},D_{i}\right)=\prod_{\tau =1}^{T}Ν\left({\dot{\phi }}_{i}(t_{\tau }\right)|{\hat{\omega }}_{i}+\sum_{j\neq i}^{N}{\hat{\Gamma }}_{i,j}(\psi_{i,j}(t_{\tau })),\frac{2D_{i}}{∆t}),$$ (11)

where ${\hat{\Gamma }}_{i,j}$ equals $\Gamma_{ij}-a_{ij}^{\left(0\right)}$, and *N*(*x*|*μ*,*σ*^2^) denotes Gaussian distribution, where *μ* and *σ*^2^ are the mean and variance of *x*, respectively. Using Bayesian theory, the product of the likelihood function and the prior distribution $p\;\left({\left\{{\hat{\omega }}_{i},a_{ij}^{\left(m\right)},b_{ij}^{\left(m\right)}\right\}}_{i},D_{i}\right)$ is proportional to the posterior distribution $p\;({\left\{{\hat{\omega }}_{i},a_{ij}^{\left(m\right)},b_{ij}^{\left(m\right)}\right\}}_{i},D_{i}|\left\{\phi_{i}(t_{\tau })\}\right)$:

$$p\;({\left\{{\hat{\omega }}_{i},a_{ij}^{\left(m\right)},b_{ij}^{\left(m\right)}\right\}}_{i},D_{i}|\left\{\phi_{i}(t_{\tau })\}\right)\propto L\;\left(\{\phi_{i}(t_{\tau })\}|{\left\{{\hat{\omega }}_{i},a_{ij}^{\left(m\right)},b_{ij}^{\left(m\right)}\right\}}_{i},D_{i}\right)\;p\;\left({\left\{{\hat{\omega }}_{i},a_{ij}^{\left(m\right)},b_{ij}^{\left(m\right)}\right\}}_{i},D_{i}\right).$$ (12)

Here, we adopt a Gaussian inverse gamma distribution for the prior distribution $p\;\left({\left\{{\hat{\omega }}_{i},a_{ij}^{\left(m\right)},b_{ij}^{\left(m\right)}\right\}}_{i},D_{i}\right)$. This prior distribution is a conjugate to the likelihood function (the so-called conjugate prior):

$$p\;\left({\left\{{\hat{\omega }}_{i},a_{ij}^{\left(m\right)},b_{ij}^{\left(m\right)}\right\}}_{i},D_{i}\right)=N\left({\left\{{\hat{\omega }}_{i},a_{ij}^{\left(m\right)},b_{ij}^{\left(m\right)}\right\}}_{i}\right|\chi_{i}^{old},\frac{2D_{i}}{∆t}\Sigma_{i}^{old})\;IG\;\left(\frac{2D_{i}}{∆t}|\alpha_{i}^{old},\beta_{i}^{old}\right),$$ (13)

where *IG*(x|α,β) denotes the inverse gamma distribution with shape parameter α and scale parameter β. $\chi_{i}^{old}$ and $\frac{2D_{i}}{∆t}\Sigma_{i}^{old}$ are the mean and covariance of model parameters $\hat{\omega },\;a^{\left(m\right)}$, and *b*^(*m*)^, respectively. Note that the prior parameters $\chi_{i}^{old},\;\Sigma_{i}^{old},\;\alpha_{i}^{old}$, and $\beta_{i}^{old}$ are referred to as hyperparameters. We can easily calculate the posterior distribution parameters from the conjugate prior distribution and the likelihood function in Eq (12) (S3 File). The posterior distribution with the updated parameters takes the form of a Gaussian inverse gamma distribution: $\chi_{i}^{new},\;\Sigma_{i}^{new},\;\alpha_{i}^{new}$, and $\beta_{i}^{new}$. The estimated model parameters are the mean of $N\;\left({\left\{{\hat{\omega }}_{i},a_{ij}^{\left(m\right)},b_{ij}^{\left(m\right)}\right\}}_{i}\right|\chi_{i}^{new},\frac{2D_{i}}{∆t}\Sigma_{i}^{new})$. The estimated noise level is the mean of $IG\;\left(\frac{2D_{i}}{\Delta t}|\alpha_{i}^{new},\beta_{i}^{new}\right)$. Using the posterior, prior, and likelihood functions, we can compute the logarithmic evidence log *p*({*ϕ*_*i*_(*t*_*τ*_)}):

$$\log p(\left\{\phi_{i}(t_{\tau })\right\})=\log\frac{L(\{\phi_{i}(t_{\tau })\}|{\left\{{\hat{\omega }}_{i},a_{ij}^{\left(m\right)},b_{ij}^{\left(m\right)}\right\}}_{i},D_{i})p({\left\{{\hat{\omega }}_{i},a_{ij}^{\left(m\right)},b_{ij}^{\left(m\right)}\right\}}_{i},D_{i})}{p({\left\{{\hat{\omega }}_{i},a_{ij}^{\left(m\right)},b_{ij}^{\left(m\right)}\right\}}_{i},D_{i}|\left\{\phi_{i}(t_{\tau })\right\})}.$$ (14)

Thus, we find *M*_*ij*_ with the largest logarithmic evidence among all models.

### Electronic circuit of van der Pol oscillator

To test the proposed method, we conducted an experiment in which an electronic circuit was used to implement two coupled van der Pol oscillators [45]. The coupling function Γ_*i*,*j*_ between the oscillators can be obtained theoretically from the corresponding differential equations.

**Fig 1:**
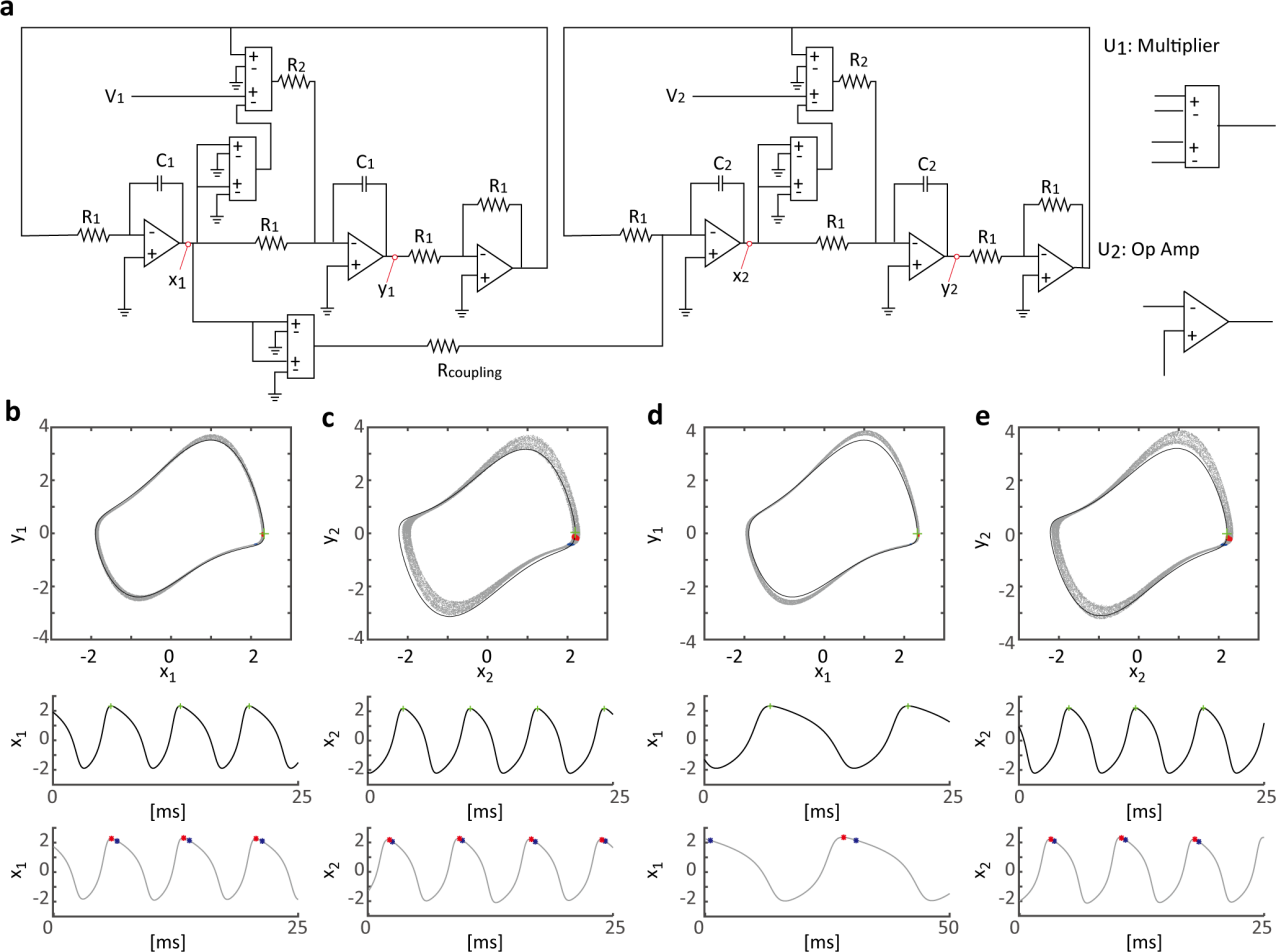
Electronic circuit of a pair of van der Pol oscillators and recorded electric potential. (a) Schematic of electronic circuit of two coupled van der Pol oscillators, where *x*_*i*_ and *y*_*i*_ are positions for recording electric potential, *R*_*k*_ denotes resistors, and *C*_*i*_ denotes condensers. Electronic units U_1_ and U_2_ represent the multiplier and operational amplifiers, respectively. *R*_*coupling*_ is a resistor whose resistance is the parameter of the strength of connectivity. (b) Experimental data of electric potentials *x*_1_ and *y*_1_ show the limit-cycle oscillator under the same-frequency (129.1 Hz) coupling condition (gray dots and line). The black trajectory shows the theoretical value computed by the van der Pol oscillator Eqs (15–18). Here, the frequency is 142.1 Hz. Blue dots represent the zero-phase reference points on the experimental data, which were determined automatically via Hilbert transformation. Green crosses represent the theoretical zero-phase reference points defined as the peak points of *x*_*i*_. Red dots denote the adjusted zero-phase reference points. (c) *x*_2_ and *y*_2_ show the oscillators under the same-frequency oscillator condition. The frequency of the experimental data is 132.5 Hz and that of the theoretical trajectory is 146.4 Hz. (d) Recorded electric potentials show the slow limit-cycle oscillator under cross-frequency coupling conditions (experimental frequency, 64.1 Hz; theoretical frequency, 71.1 Hz). (e) *x*_2_ and *y*_2_ denote the fast oscillator (experimental frequency, 131.1 Hz; theoretical frequency, 146.4 Hz).

In this experiment, we recorded the rhythmic signals of the electronic circuit. Each oscillator consisted of two multipliers U_1_ (AD633, Low Cost Analog Multiplier) and three operational amplifiers U_2_ (TL082, ½ Dual BiFET Op Amp) (Fig 1A). We conducted two experiments under different conditions. In the first experiment, two same-frequency oscillators were coupled directly. In this experiment, 1:1 phase locking was expected to occur. The parameters of the electronic component were set to *R*_1_ = 100kΩ, *R*_2_ = 1kΩ, *R*_*coupling*_ = 1MΩ, *C*_1_ = *C*_2_ = 0.01μF, *V*_1_ = 0.115V, and *V*_2_ = 0.12V. R_*k*_ and C_*i*_ are the parameters of the resistor and capacitor, respectively. Voltages *V*_*i*_ were monitored using a digital voltmeter. In the second experiment, a slow oscillator was coupled to a fast oscillator. In this experiment, 1:2 phase locking was expected to occur. The natural frequencies of the slow and fast oscillators were set to satisfy a nearly 1:2 ratio. The parameters of the electronic components were the same as those in the first experiment, except that *C*_1_ was changed to *C*_1_ = 0.02μF to reduce the natural frequency by one-half. In both cases, the electric potentials x_*i*_ and y_*i*_ were recorded using an I/O device (NI SCB-68, National Instruments, US). The sampling rate of the electric potential was 15,000 Hz, and the data size was 180 s.

The corresponding theoretical equation of the electronic circuit is given as:

$$\frac{dx_{1}}{dt}=\frac{1}{R_{1}C_{1}}y_{1},$$ (15)

$$\frac{dy_{1}}{dt}=\frac{1}{{100R}_{2}C_{1}}(10V_{1}-x_{1}^{2})y_{1}-\frac{1}{R_{1}C_{1}}x_{1},$$ (16)

$$\frac{dx_{2}}{dt}=\frac{1}{R_{1}C_{2}}y_{2}-\frac{1}{{10R}_{coupling}C_{2}}x_{1}^{2},$$ (17)

$$\frac{dy_{2}}{dt}=\frac{1}{{100R}_{2}C_{2}}(10V_{2}-x_{2}^{2})y_{2}-\frac{1}{R_{1}C_{2}}x_{2}+\frac{1}{R_{1}C_{2}}b,$$ (18)

where *x*_*i*_ and *y*_*i*_ are the corresponding theoretical electric potentials of the *i*-th oscillator. The trajectories of the van der Pol oscillator are shown by the *x*_*i*_ and *y*_*i*_ signals in Fig 1B–1E. Note that only *x*_*i*_ was used to estimate the coupling function. The experimental parameters (R_k_ and *C*_*i*_) and those in Eqs (15–18) were the same as the parameters of the electronic components. Note that the term *b* does not exist in the original van der Pol oscillator. In the case of *b* = 0, the theoretical trajectory and coupling function do not agree with the experimental data and the estimated coupling functions, respectively. Note that the original van der Pol oscillator generates a symmetrical trajectory. However, in the electronic circuit experiment, the trajectory was not exactly symmetrical due to small additional disturbances in the system or the uneven quality of the electronic components. By introducing parameter *b* (*b* = 0.4), the simulated trajectory can be adjusted to the experimental data (Fig 1B–1E).

The corresponding theoretical coupling functions were calculated based on Eqs (15–18) using the adjoint method [46]. In this method, the zero phase is defined as the peak point of *x*_*i*_ (green crosses in Fig 1B–1E represent the zero-phase reference points on the theoretical orbits). From the experimental data, the zero-phase reference points were determined based on electric potentials *x*_*i*_ using Hilbert transformation (blue dots denote the point of zero-phase reference on the experimental data). As shown in Fig 1B–1E, a small gap exists between the zero-phase reference points of the theoretical and experimental orbits. In principle, an arbitrary point on the limit-cycle orbit can be defined as the zero-phase point. However, to compare theoretical and estimated results, the zero-phase reference point of the theoretical model should be consistent with that of the experimental data. Therefore, the zero-phase reference points of the experimental orbits were adjusted to coincide with those of the theoretical model by shifting the experimental reference points to the theoretical points as $\phi_{1}=\phi_{1}+\frac{\pi }{6},\;\phi_{2}=\phi_{2}+\frac{\pi }{10}$ (red dots denote the revised zero-phase reference points), where *ϕ*_1_ is the experimental phase based on the electric potential *x*_1_ (Fig 1B and 1D) and *ϕ*_2_ is the experimental phase based on the electric potential *x*_2_ (Fig 1C and 1E). We estimated the coupling function from the shifted phase data and compared the theoretical coupling function with the estimated function.

### Scalp EEG experiment

We applied the proposed method to scalp EEG data. The method can estimate the coupling functions for same-frequency and cross-frequency synchronization assuming that EEG activities can be considered weakly coupled oscillators. However, it is unclear whether EEG activity can be considered a weakly coupled oscillator system. Thus, we must confirm that the proposed method can estimate the dynamical system from the EEG data successfully.

We used EEG data recorded during a speech recognition task. Note that detailed information is provided in our previous paper [37]. The participants categorized what they heard as a target or distractor as soon and as accurately as possible. Four-letter Japanese words were used as speech sounds, and the words were uttered within approximately 1 s. The sampling rate of the speech sound was 48,000 Hz. The speech envelopes on each frequency were high-pass filtered with a cutoff frequency of 3 Hz to avoid phase-resetting. Furthermore, the speech sounds were masked with pink noise. The noise volume was increased linearly over 0.5 s after onset to avoid the phase-resetting effect by noise onset. The speech sound always started 2 s after the onset of noise and lasted approximately 1 s. The noise sound was terminated 1.5 s after speech onset. The EEG experiment consisted of four sessions for each participant. Each session consisted of 100 trials.

A 32-channel EEG amplifier (Brain Amp MR, Brain Products, Germany) with an international 10% standard electrode cap with a sintered Ag/AgCl ring electrode (Easy Cap, Falk Minow Services, Germany) was used for the EEG recording (sample rate, 5 kHz). Four electrodes were used for the vertical and horizontal electrooculogram (VEOG and HEOG) channels. The VEOG and HEOG were used to remove ocular artifacts. The measurement reference was linked earlobes, and the ground was on the inion. The EEG signal was filtered using a 1-Hz high-pass software filter, a 250-Hz low-pass hardware filter, and a 60-Hz notch filter. In a preprocessing step, ocular artifacts were corrected using EEG analysis software (Brain Vision Analyzer, Brain Products, Germany) and the VEOG/HEOG signals [47]. The reference was changed to the average of all electrodes, except VEOG and HEOG. The preprocessed EEG data were then downsampled to 500 Hz.

The participants were 16 healthy adults (five females; 11 males; 21–32 years; mean age, 25 years). One participant was excluded due to a low response rate, and another participant was excluded due to an excessive artifact that could not be removed during preprocessing. Note that these participants were also excluded in our previous study [37].

**Fig 2:**
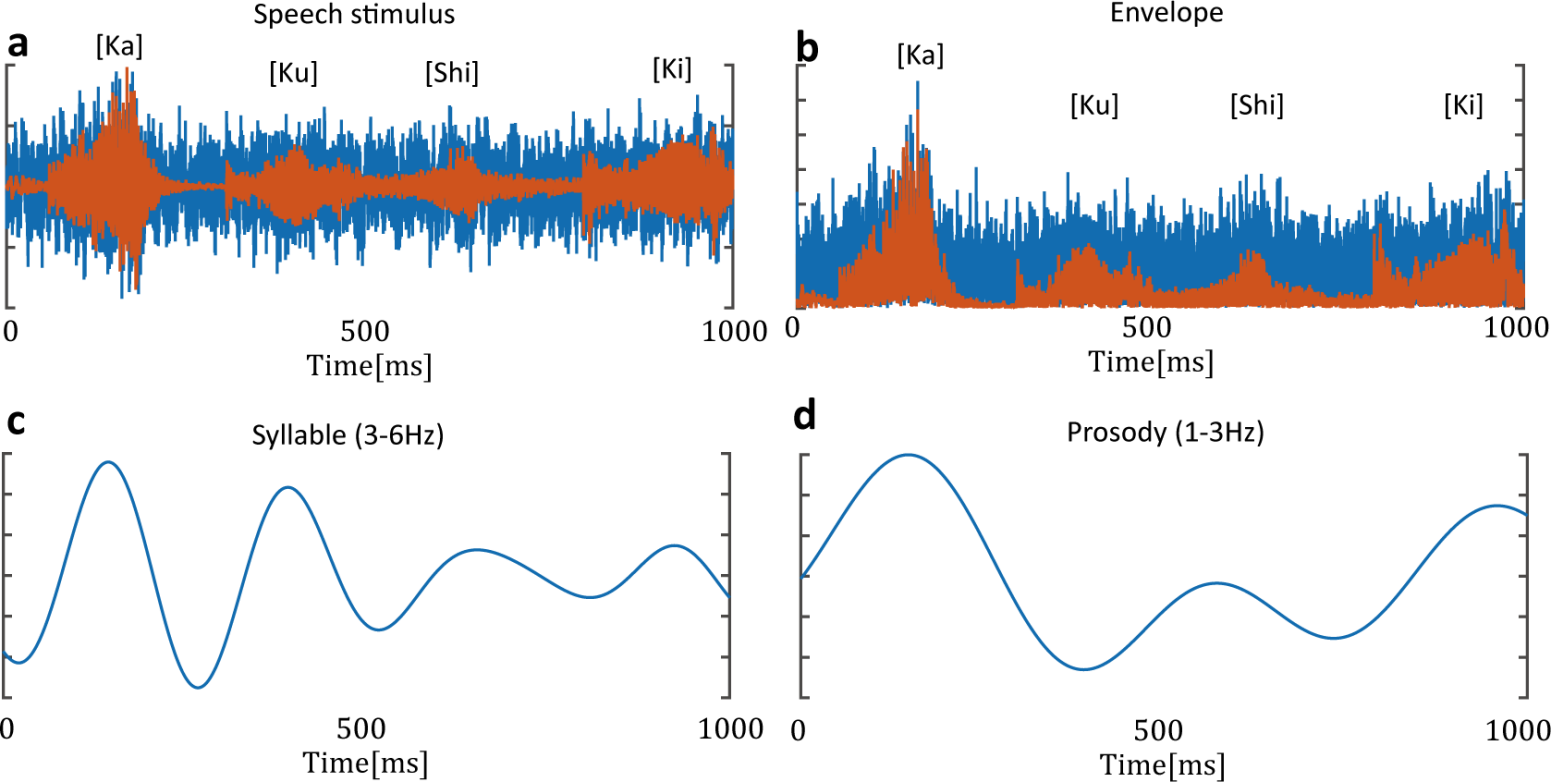
Syllable and prosody rhythms in speech sound. (a) Example of speech stimulus. The stimulus consisted of noise and a four-syllable Japanese word. The red line represents a speech wave. The blue line represents the presented sound wave, which consists of speech plus noise sounds. (b) Speech envelope was computed as the absolute value of Hilbert-transformed speech sound. (c) Syllabic rhythms were computed from the speech envelope through the bandpass filter within 3–6 Hz. (d) Prosodic rhythms were computed from the speech envelope through the bandpass filter within 1–3 Hz.

We estimated the phase oscillator model between the 3–6 Hz EEG (theta oscillation) and the speech envelope. The theta oscillation is synchronized with the envelope of speech sound, and it plays an important role in speech processing [35, 48, 49]. Speech rhythm consists of linguistic components, e.g., syllabic and prosodic rhythms. A syllable is a unit of speech that separates a word into sound chunks. For example, the Japanese word “KaKuShiKi” (“formality” in English) is composed of four syllables “/Ka/Ku/Shi/Ki/,” and its sound envelope appears in the 4–5 Hz frequency range in the current speech stimulus. Prosody is the stress and intonation patterns of an utterance. The envelope of prosodic rhythms appears in the 1–3 Hz frequency range. We estimated the coupling function between the EEG oscillation and these speech rhythms. To obtain the theta oscillations, the preprocessed EEG signals were bandpass filtered within 3–6 Hz. The syllabic and prosodic rhythms were computed from the sound stimulus consisting of the noise and speech sounds (Fig 2A). The sound envelope was computed as the absolute value of the Hilbert-transformed sound data (Fig 2B). To compute the syllabic and prosodic rhythms, the envelope signal was bandpass filtered within 3–6 Hz and 1–3 Hz, respectively (Fig 2C and 2D). The syllabic and prosodic signals were downsampled to 500 Hz. The instantaneous phases of these rhythms were computed using both Hilbert transformation and correction (Eq 8). We estimated the coupling functions of 1:1 phase locking (syllable and theta oscillation) and 1:2 phase locking (prosody and theta oscillation).

## Results

### Numerical simulation of phase oscillator model

**Fig 3:**
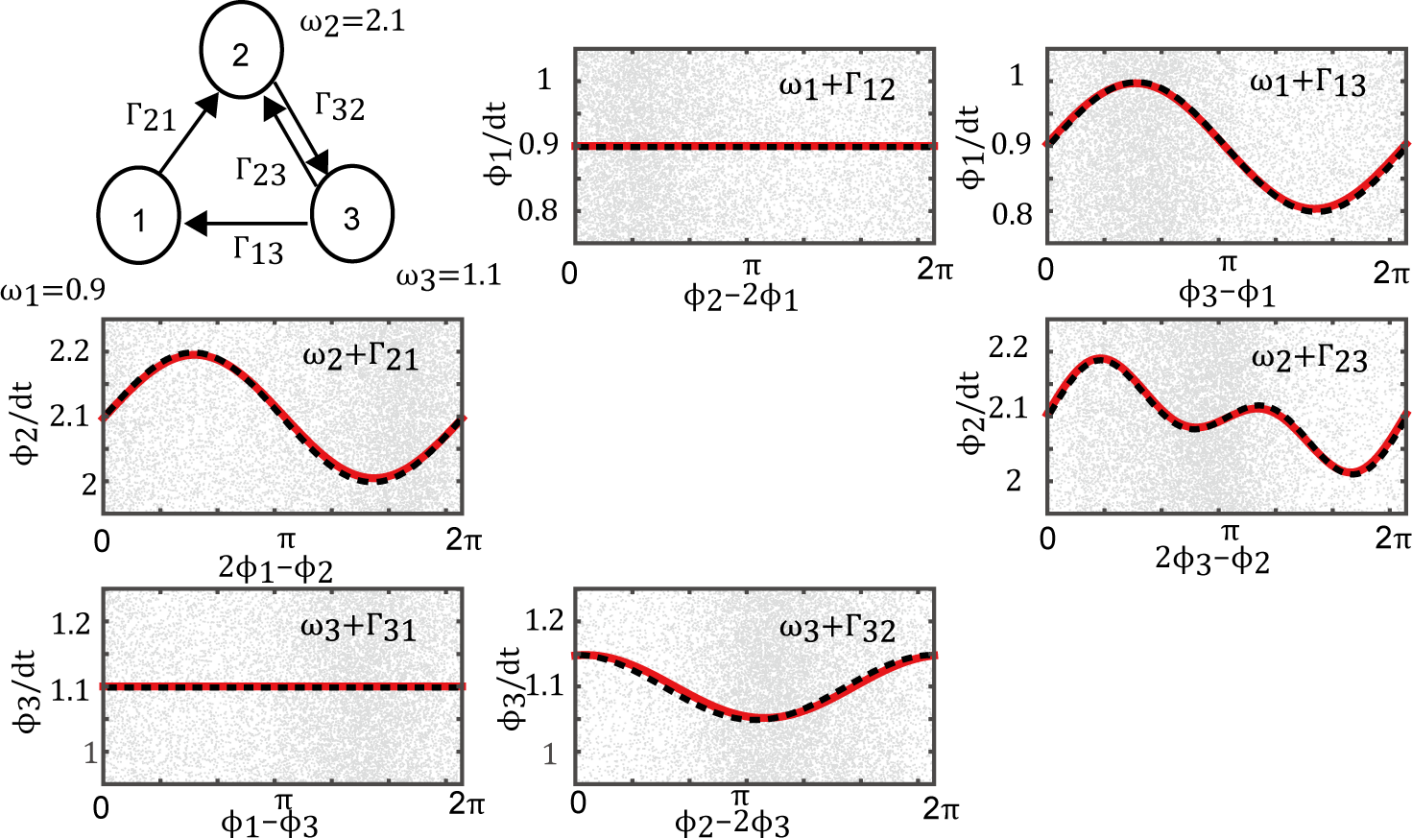
Estimated coupling function for numerical simulation data. Upper-left diagram shows the network structure. The estimated coupling functions (red lines) were nearly identical to the correct functions (dashed black line). The gray dots represent the phase time-series data. When the interaction did not exist, the estimated coupling function was identically zero. The proposed method estimated all coupling functions correctly for the simulation data.

First, we applied our Bayesian method to numerical simulation data which was generated from three cross-frequency oscillators with somewhat complicated connections, to evaluate the validity of the proposed method. Simulation data were generated from a network comprising one fast oscillator and two slow oscillators (Fig 3) based on the Euler–Maruyama method [50] using the following differential equations:

$$\frac{d\phi_{1}}{dt}=\omega_{1}+0.1sin(\phi_{3}-\phi_{1})+\eta_{1}(t),$$ (19)

$$\frac{d\phi_{2}}{dt}=\omega_{2}+0.1sin({2\phi }_{1}-\phi_{2})+0.05\{sin({2\phi }_{3}-\phi_{2})+sin({2(2\phi }_{3}-\phi_{2}))\}+\eta_{2}(t),$$ (20)

$$\frac{d\phi_{3}}{dt}=\omega_{3}+0.05cos(\phi_{2}-2\phi_{3})+\eta_{3}(t).$$ (21)

We set the natural frequencies to *ω*_1_ = 0.9, *ω*_2_ = 2.1, and *ω*_3_ = 1.1. Here, *ϕ*_2_ is the phase of the fast oscillator, and *ϕ*_1_ and *ϕ*_3_ are the phases of the slow oscillators. *η* is independent Gaussian white noise with zero mean and a standard deviation of 0.1. Using the proposed method, we estimated the coupling functions and the natural frequencies from the phase time-series data generated in the experiment.

Fig 3 shows the estimated coupling functions and the correct coupling functions. In this case, the correct coupling functions were defined explicitly by Eqs (19)–(21). Despite the complicated connections, the results indicate that the estimated and correct coupling functions agree fairly well. Furthermore, the complexity parameter of the coupling function was selected correctly by optimizing the logarithmic evidence. Therefore, the proposed method works quite well at estimating a nontrivial network of phase oscillators comprising oscillators with different natural frequencies.

### Electronic circuit experiment

**Fig 4:**
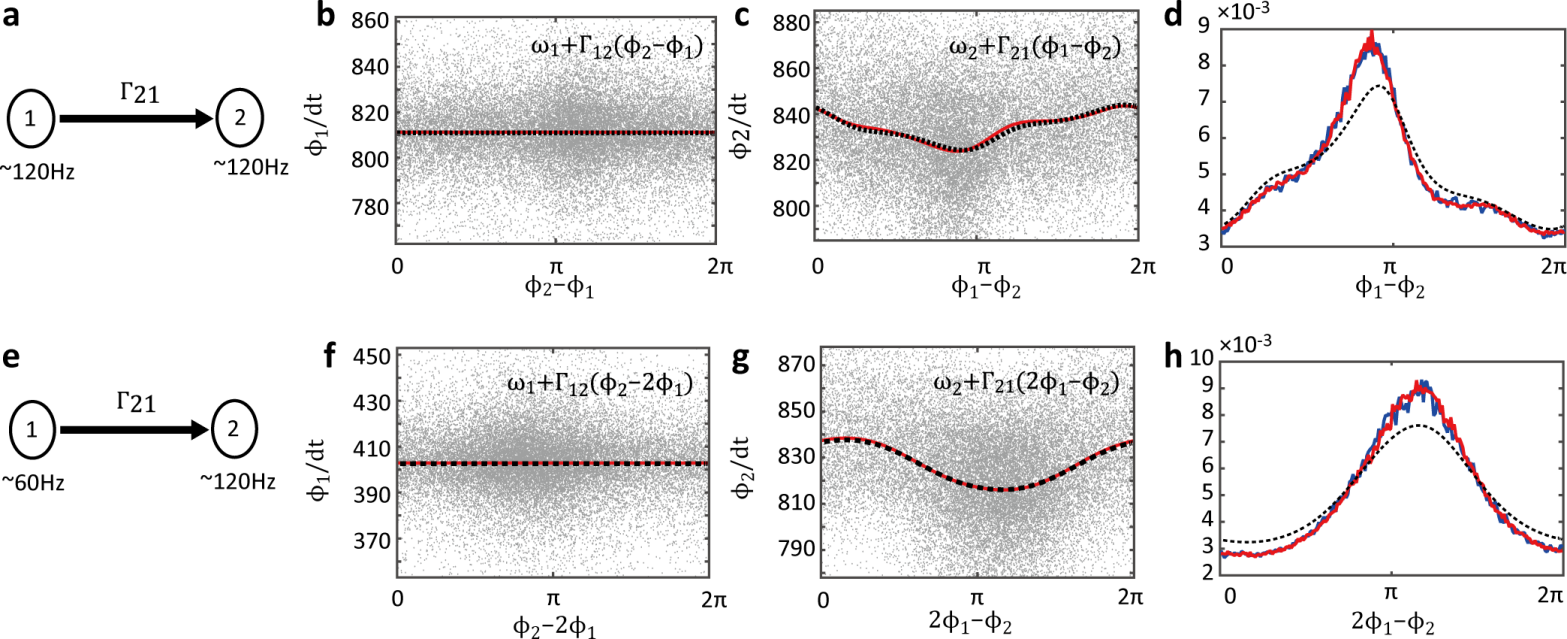
Estimated coupling function of electronic circuit. (a) The diagram shows the coupling direction between oscillators of the same frequency. The first oscillator was coupled to the second oscillator. (b) The red line shows the estimated phase coupling function with the natural frequency in the same-frequency coupling case. The dashed black line shows the theoretical coupling function. The coupling function from the second to first oscillator Γ_12_ is identically zero. When there is no interaction, the coupling function is nearly zero. The gray dots show the experimental data points. (c) The coupling functions from the first to second oscillator Γ_21_. (d) The blue line shows the phase difference histogram of the experimental data in the case of 1:1 phase locking (experimental histogram). The red line shows the simulated histogram calculated in the phase oscillator model estimated from the experimental data (estimated histogram). The dashed black line shows the simulated histogram calculated in the phase oscillator model using the theoretical natural frequencies and coupling functions (theoretical histogram). (e) In the cross-frequency coupling case, the slow oscillator was coupled to the fast oscillator. (f) The coupling function from the fast to slow oscillator is identically zero. (g) The coupling function from the slow to fast oscillator. (h) The experimental, estimated, and theoretical histogram in the 1:2 phase-locking case.

Before applying the proposed method to EEG data, we recorded the electric potential of the van der Pol electronic circuit and tested the ability of the estimation method using the experimental data. Since the electronic circuit can be explained by the corresponding theoretical differential equations (Eqs 15–18), we can derive the correct coupling function theoretically using the adjoint method. We conducted two experiments. One involved coupling oscillators of the same frequency (Fig 4A), and the other involved coupling slow and fast oscillators (Fig 4E). We transformed the *x*_1_ and *x*_2_ electric potentials of the first and second oscillators, respectively, to phase time-series data and estimated the coupling functions from these data.

The estimated coupling functions with no interaction from the second to first oscillator were identically zero in the experiments involving oscillators of the same frequency (Fig 4B) and cross frequency (Fig 4F). When coupling existed, the estimated coupling function was the same as the theoretical function under the same-frequency (Fig 4C) and cross-frequency conditions (Fig 4G). Furthermore, to confirm whether the estimated phase oscillator model can explain the experimental data, we compared the phase difference histograms for the experimental phase data and the two types of simulated phase data. The experimental phase data were computed from electric potentials by both Hilbert transformation and Eq (8). One set of simulated phase data was computed in the phase oscillator model based on the estimated parameters using the Euler–Maruyama method. The other was computed based on the theoretical coupling functions and natural frequency, which were determined by Eqs (15)–(18). We computed the experimental, estimated, and theoretical histograms from these phase time-series data. The experimental and estimated histograms of the phase difference were nearly the same under each condition (Fig 4D and 4H). However, the theoretical histograms differed. The difference among these histograms was caused by the difference of the natural frequency between the theoretical and experimental oscillators (Fig 1B–1E) due to uncontrollable experimental conditions. In other words, the electronic circuit experimental data did not follow the theoretical equations exactly; however, the coupling functions between the experimental van der Pol oscillators were the same as the theoretical coupling functions. In fact, when the theoretical histograms were computed based on the theoretical coupling functions and the estimated natural frequencies rather than the theoretical natural frequencies, the experimental and estimated histograms coincided relatively well with the theoretical histograms. These results indicate that the proposed method works well with real data, even if the data contain observational errors or additional unknown disturbances.

### Human EEG experiment

**Fig 5:**
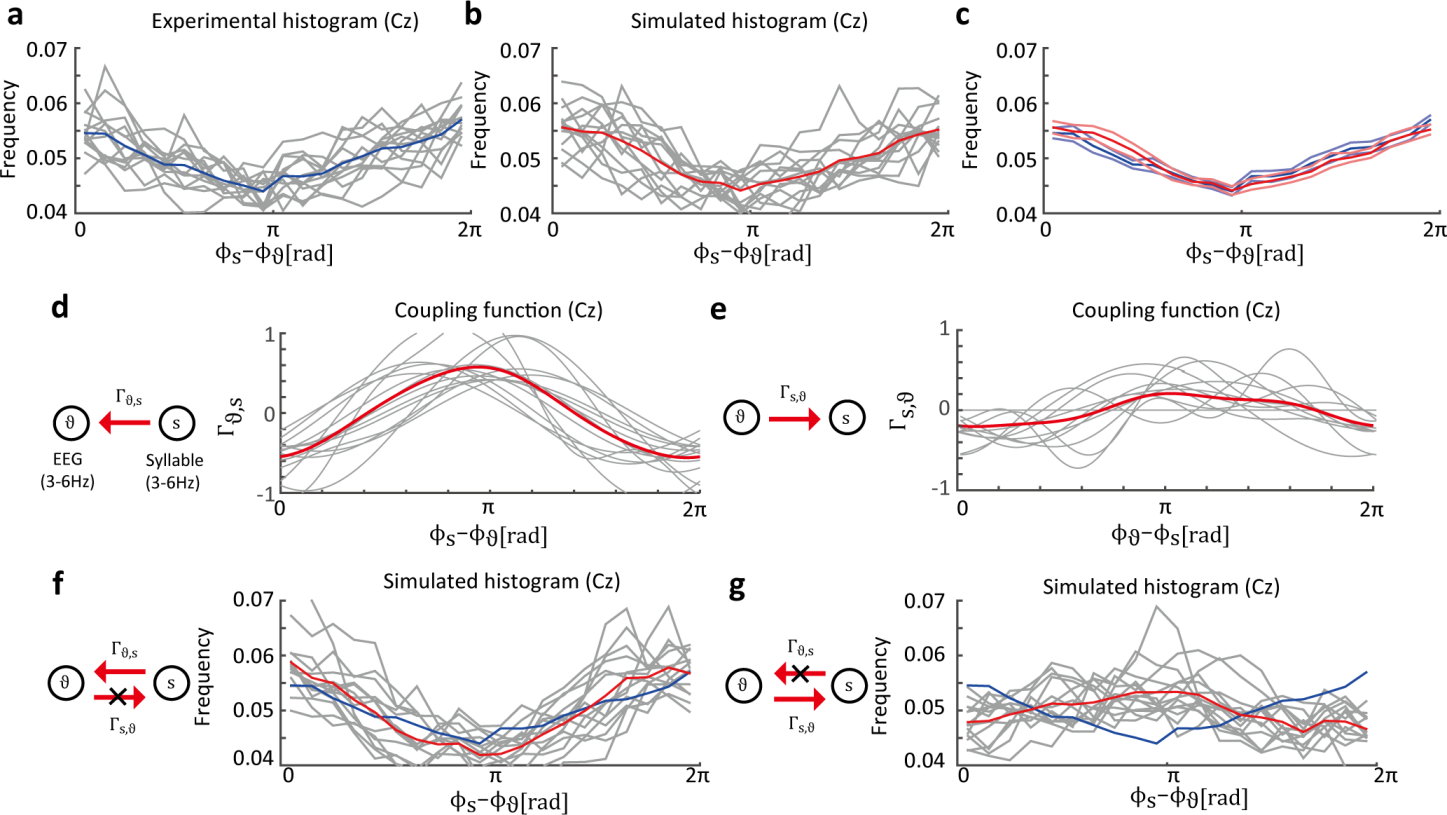
Estimated distribution of phase difference between EEG data and syllable envelope. (a) Experimental histogram of phase difference between the theta oscillation on the Cz electrode and the syllabic rhythm (the histograms show phase locking). The gray lines represent histograms of individual participants and the blue line represents the histogram averaged over all participants. (b) Histograms obtained from the simulated data in the estimated phase oscillator model. The averaged histogram is similar to the experimental histogram. The gray lines represent the phase difference histograms of individual participants. The red line represents the average of the simulated histograms. (c) Blue lines represent the averaged experimental histogram and the standard error of mean (SEM). Red lines represent the averaged simulated histograms and the SEM. (d) Estimated coupling functions Γ_*θ*,*s*_ from syllabic rhythm to theta oscillation. The gray and red lines represent the results of individual participants and the average results of all participants, respectively. (e) Estimated coupling functions Γ_*s*,*θ*_ are considerably smaller than the opposite directional coupling functions. (f) Simulated histograms where the coupling functions Γ_*s*,*θ*_ are removed. The effect on the original phase-locking state was negligible. (g) Histograms where Γ_*θ*,*s*_ were removed are nearly flat.

**Fig 6:**
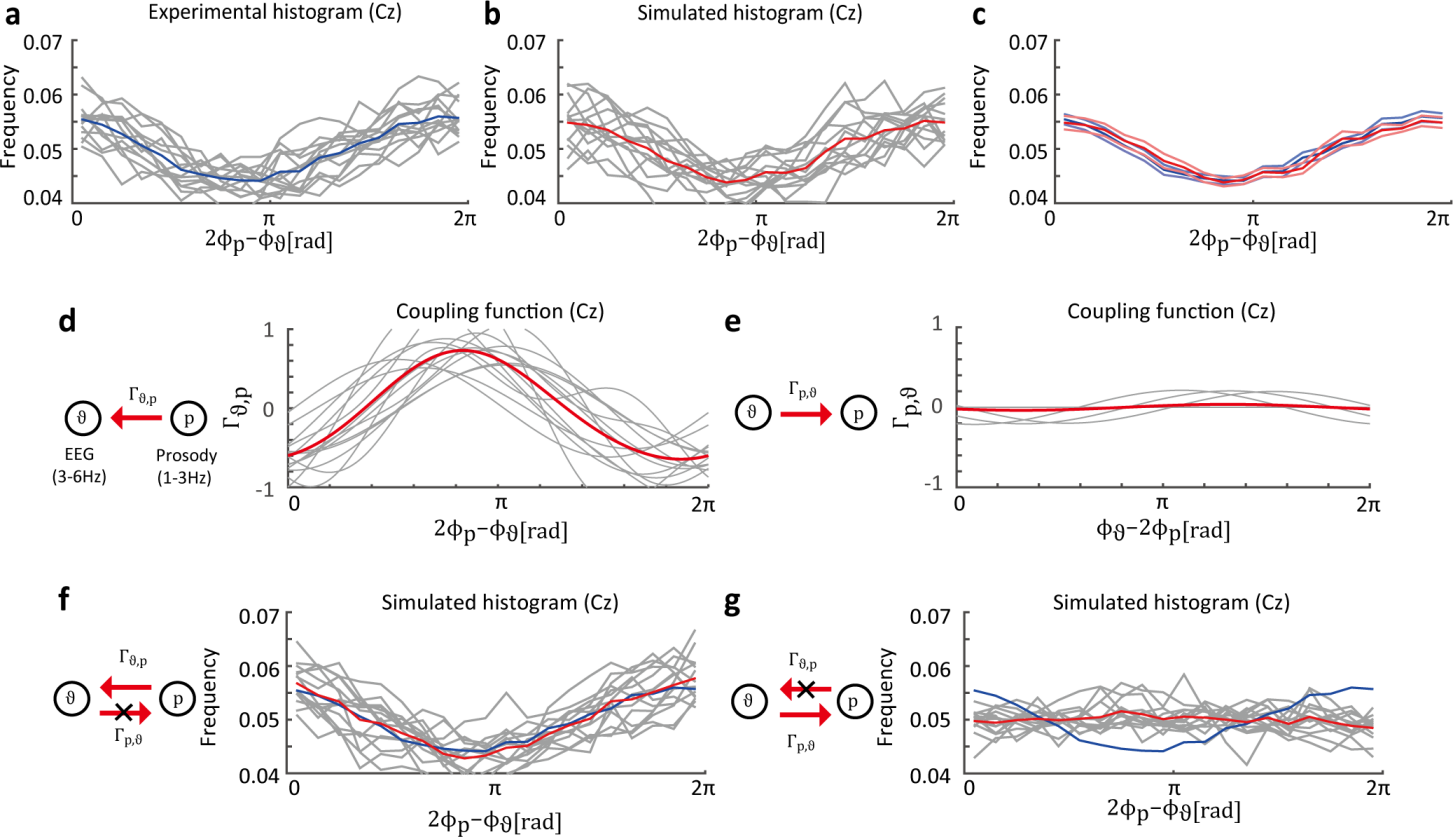
Estimated distribution of phase difference between EEG data and prosody envelope. (a) Experimental phase difference histograms for 1:2 phase locking. (b) Simulated histograms based on the estimated phase oscillator model. (c) Blue lines represent the average and SEM of experimental phase difference histograms. Red lines represent the average and SEM of simulated histograms. (d) Estimated coupling functions Γ_*θ*,*p*_. (e) Estimated coupling functions Γ_*p*,*θ*_. (f) Simulated histograms where coupling functions Γ_*p*,*θ*_ are removed. (g) Simulated histograms where Γ_*θ*,*p*_ is removed are uniform.

Finally, we applied the proposed method to the EEG data. We estimated the coupling functions between the theta oscillation and the envelope of speech stimulus. The theta oscillation was observed to be synchronized with the speech envelope [35] and is considered to play an important role in speech perception. Generally, the speech envelope consists of syllabic (3–6 Hz) and prosodic (1–3 Hz) rhythms. Thus, we estimated the phase oscillator model under the same-frequency and cross-frequency conditions, i.e., theta oscillation and syllabic rhythm (Fig 5), and theta oscillation and prosodic rhythm (Fig 6). In these experiments, the phase-difference histograms between the EEG and the speech sound showed 1:1 and 1:2 phase locking (Figs 5A and 6A). Note that there is obviously no interaction from EEG activity to speech sound. Therefore, we can use this fact to confirm the validity of the estimation results.

In the theta oscillation and syllable data, we assumed that the syllabic rhythm modulated the theta oscillation. The instantaneous phase of the theta oscillation is denoted *ϕ*_*θ*_, and the phase of the syllable is denoted *ϕ*_*s*_. The phase difference between the theta oscillation and the syllable is defined as *ϕ*_*s*_ − *ϕ*_*θ*_. The phase-difference histogram of each participant showed 1:1 phase locking (Fig 5A). We estimated the phase oscillator models of 1:1 phase locking as follows:

$$\frac{d\phi_{\theta }}{dt}=\omega_{\theta }+\Gamma_{\theta ,s}(\phi_{s}-\phi_{\theta })+\eta_{\theta }(t),$$ (22)

$$\frac{d\phi_{s}}{dt}=\omega_{s}+\Gamma_{s,\theta }(\phi_{\theta }-\phi_{s})+\eta_{s}(t).$$ (23)

To confirm that the estimated dynamical system can explain the experimental data, we simulated phase synchronization based on the estimated phase oscillator model. Fig 5B shows the phase difference histograms obtained from the simulated data. Note that, for the simulated data, phase differences were calculated by numerical simulation performed using the estimated phase oscillator model (Eqs (22 and 23)). The histograms of the simulation data were similar to those of the experimental data (Fig 5C), which indicates that the estimated phase oscillator model can explain the experimental data. Furthermore, the estimated coupling functions Γ_*θ*,*s*_ were consistent among all participants (Fig 5D). In contrast, the estimated coupling functions Γ_*s*,*θ*_ had smaller amplitudes than Γ_*θ*,*s*_ (Fig 5E) and were not consistent among all participants. These results are reasonable in terms of the relationship between EEG and speech sound because direct interaction from theta oscillation to speech sound never exists. To examine the effects of each coupling function on the dynamics, we computed the simulated histogram under the condition that either Γ_*s*,*θ*_ or Γ_*θ*,*s*_ was set to identically zero. In the case of Γ_*s*,*θ*_ = 0 (Fig 5F), the resultant histogram shows that, compared to the original dynamics in Fig 5B, the synchronized state is almost maintained. This implies that the coupling function Γ_*s*,*θ*_ does not contribute to the realization of 1:1 phase locking. In the case of Γ_*θ*,*s*_ = 0, the synchronized state disappeared, as shown in the flatter histograms (Fig 5G). Consequently, the results indicate that the coupling function Γ_*θ*,*s*_ primarily contributed to 1:1 phase locking.

In the theta oscillation and prosody data, we assumed that the prosodic rhythms modulated the theta oscillation. Here, let *ϕ*_*p*_ denote the prosody phase. Considering the 1:2 phase-locking state, the phase difference between theta oscillation and prosody is reasonably defined as 2*ϕ*_*p*_ − *ϕ*_*θ*_. The phase difference histograms of each participant showed 1:2 phase locking (Fig 6A). Next, we considered the phase oscillator model for 1:2 phase locking as follows:

$$\frac{d\phi_{\theta }}{dt}=\omega_{\theta }+\Gamma_{\theta ,p}(2\phi_{p}-\phi_{\theta })+\eta_{\theta }(t),$$ (24)

$$\frac{d\phi_{p}}{dt}=\omega_{p}+\Gamma_{p,\theta }(\phi_{\theta }-{2\phi }_{p})+\eta_{p}(t).$$ (25)

We confirmed that the estimation result can explain the experimental data by calculating the simulated phase difference histograms under the 1:2 phase-locking condition (Fig 6B). The simulated histograms were similar to the experimental histograms (Fig 6C). The results indicate that the estimation phase interaction functions can explain the experimental data, as well as the 1:1 phase-locking condition. The estimated coupling functions of all participants were consistent (Fig 6D and 6E). Furthermore, the estimated coupling functions Γ_*p*,*θ*_ showed small amplitude or were identically zero (Fig 6E). These results clearly show that there were no coupling function from EEG to speech sound, which is reasonable given the relationship between EEG and speech sounds in the experiments. In the case of Γ_*p*,*θ*_ = 0, the simulated phase difference histograms also showed phase locking (Fig 6F), as well as Fig 6B. In contrast, phase locking disappeared for Γ_*θ*,*p*_ = 0 (Fig 6G). These results indicate that the coupling function Γ_*θ*,*p*_ contributed to 1:2 phase locking.

In both cases, our method could estimate whether there was a relationship between the EEG activity and speech sound even though there was some variance due to estimation inaccuracies. In the case of the theta oscillation and prosody data, the estimated coupling functions Γ_*p*,*θ*_ showed small amplitudes or were identically zero, clearly demonstrating the asymmetry of the relationship between the EEG activity and speech sound. In contrast, for the theta oscillation and syllable data, the estimated coupling functions Γ_*s*,*θ*_ showed somewhat larger amplitudes than Γ_*p*,*θ*_, giving no clear indication of an asymmetric relationship. Therefore, in order to determine whether there was a asymmetry relationship, we estimated the coupling functions using surrogate data (S1 Fig). The surrogate data consisted of randomly time-shifted speech-sound phase data and original EEG data that had not been time-shifted. Note that there was no temporal relationship between the time-series, speech-sound phase data and the original EEG signals. This random shifting process was repeated 100 times for each of the 14 participants. Then, we estimated the coupling functions using these 1,400 surrogate datasets and computed histograms of the model selection results for the appropriate Fourier modes based on the logarithmic evidence and the coupling function powers $\int_{0}^{2\pi }{\left|\Gamma (\psi )\right|}^{2}d\psi$. For the coupling functions Γ_*θ*,*s*_ and Γ_*θ*,*p*_, the model selection histograms showed that the M = 0 model was selected more often than the other models (S1A and S1E Fig), while the original data results showed that none of the participants selected the M = 0 model. Furthermore, the integrated values of coupling function power showed that the original data results did not follow the same histograms as the surrogate data results (S1B and S1F Fig). For the coupling functions Γ_*s*,*θ*_ and Γ_*p*,*θ*_, the model selection results for the original data were not largely different from those that for the surrogate data (S1C and S1G Fig), and the integrated coupling function values were relatively small (S1D and S1H Fig). Consequently, these results suggest that the coupling functions, Γ_*s*,*θ*_ and Γ_*p*,*θ*_, showed no relationship between the EEGs and speech sounds.

## Discussion

We have proposed an estimation method to identify the phase dynamics of cross-frequency synchronization using rhythmic time-series data. By identifying the dynamics, we can reveal the direction of coupling and the role of each coupling function in synchronization. To confirm the reliability of the proposed method, we estimated a dynamical system from time-series data obtained by numerical simulation and experimentation using an electronic circuit, and we showed that these results were estimated successfully. In addition, we applied the proposed method to scalp EEG data and evaluated its validity based on the estimation results.

### Validity of estimation method for cross-frequency synchronization

We can obtain the theoretical coupling function from the numerical simulation and data from the electronic circuit experiment. To confirm the reliability of the proposed method, we compared the estimated results to theoretical results. In the simulation, time-series data were generated by numerial simulation used in the given phase oscillator model. In this case, we knew the true instantaneous phase of the time-series data and the correct coupling functions. The proposed method worked well with the simulation data (Fig 3), and the complexity parameter for the coupling function M_*ij*_ was selected correctly.

We also estimated a dynamical system using electric potential data, i.e., real time-series data. In this situation, the corresponding theoretical coupling function was computed using the adjoint method from which we constructed a theoretical model of real electronic circuits. The results demonstrated that the proposed method can correctly estimate the coupling functions (Fig 4). Furthermore, to confirm that the estimated phase oscillator model can reproduce real time-series data, we compared the phase difference histogram of real time-series data with those of the simulated data. The results demonstrate that the estimated coupling functions and the noise strengths can explain the real data, including any additional disturbance imposed on the system.

In the EEG data, the correct coupling function to be compared to the estimated function is unknown. Therefore, we must consider an alternative procedure to examine the validity of the estimation results. To this end, we considered the following three steps. In the first step, we focused on the coupling functions from the EEG activity to the speech stimulus (Figs 5E and 6E). Under this EEG experimental condition, EEG activity did not influence speech sound because the timing of the external speech sound was given by a recorded sound. Our estimated dynamics showed that the coupling function from EEG to speech sound had smaller amplitude than the coupling function in the opposite direction (Figs 5D and 6D) and did not influence the phase difference histograms (Figs 5F and 6F). Therefore, the estimated network structure is consistent with the real EEG and speech system under this experimental condition. In the second step, we confirmed whether the simulated phase difference histograms were similar to the experimental histograms. Our estimation results and the experimental data both showed phase locking (Figs 5A, 5B, 6A and 6B). In addition to results of the first step, these results suggest that the phase oscillator model can explain the EEG and speech sound data. In the final step, we confirmed that the estimated coupling functions were consistent across all participants to check whether the above results occurred by chance. Our results indicated that the coupling functions and phase difference histograms were similar across all participants on the Cz electrode (Figs 5 and 6). Furthermore, the estimation results for neighbor electrodes (e.g., FCz, Pz, CP, and CP2) showed results similar to those obtained on the Cz electrode. Based on the results obtained by performing these three steps, the mechanism between EEG and speech sound can be explained by the dynamical phase oscillator system.

### Remarks on estimation method

To apply the proposed method to EEG data, it is necessary to consider whether the systems to be estimated can be considered a weakly coupled oscillator system. It is well known that EEG phases are often locked by external input among trials [51, 52]. In addition, it is possible that phase locking is generated by phase-resetting through strong external inputs. If phase-resetting depends on the timing of external inputs rather than the phase difference, the interaction between the EEG phase and the stimulus cannot be explained by the phase oscillator model. Therefore, it is necessary to avoid phase resetting caused by sudden and strong external inputs. Event related phase locking is often induced at stimulus onset. Therefore, to prevent such phase-resetting, we presented noise (increased linearly over 0.5 s) prior to presenting speech sound. Furthermore, to avoid a situation where a strong external input induces phase-resetting at speech onset, we employed a bandpass filter to decrease the strong periodic speech sound signals.

Note that the proposed method cannot estimate the coupling function if the EEG phase is completely synchronized. Under such synchronization conditions, each phase difference between the two oscillators takes only a specific value. Therefore, except for this specific value, there is no information about the coupling function on the other phase difference value. To obtain the full range of the coupling function, the phase differences in the data must be distributed in the range 0 to 2π, as shown in Fig 3.

This study focused primarily on 1:*p* phase synchronization; however, other types of cross-frequency synchronization exist [8, 10], e.g., phase-amplitude, amplitude-amplitude, and phase-frequency synchronization. These synchronizations are also important from a cognitive function perspective; however, the proposed method cannot be applied to such dynamical systems. In future, we plan to construct a method that is applicable to the experimental data of these synchronizations.

Methods to quantify the causality between different frequency rhythms [53–56] have been proposed. Such methods, including transfer entropy and Granger causality, may reveal more general causality than the proposed method, which can only estimate the coupling function related to the *p*_*i*_:*p*_*j*_ phase synchronization. However, the proposed method can reveal causality and quantify the phase interaction function; thus, it can examine the role of connection in phase synchronization from a dynamical system perspective.

The proposed method can estimate the coupling functions of simulation data and experimental electronic circuit data accurately. In the EEG experiment, we estimated the dynamical system of EEG and speech sound. Note that we examined the dynamics of phase synchronization between a single EEG activity and speech sound rather than between two EEG activities. We applied the proposed method to synchronization between EEG and speech because the direction of causality between EEG and speech sound is clear, whereas that of EEG phases is unknown. Therefore, EEG and speech data were used to verify the estimation results. It is expected that the proposed method can serve as a useful tool to reveal the role of connectivity and causality in neural oscillations.

## Supporting information

S1 File Estimated resultant data of theta oscillation and syllabic rhythm. (ZIP)

S2 File Estimated resultant data of theta oscillation and prosodic rhythm. (ZIP)

S3 File Hyperparameters of prior distribution and updating rule. (PDF)

S1 FigHistograms of property of estimated coupling functions in surrogate data.We estimated the coupling functions for surrogate data which have no temporal relationship between the EEGs and speech sounds for comparing the estimated results with the original data. The surrogate data consisted of the original EEG phases and time-shifted speech sound phases. The speech sound phases were randomly time-shifted for each trial so that there would be no temporal relationship between the EEG and speech sound phases. This random shifting process was repeated 100 times for each of the 14 participants. We then estimated the coupling functions using these 1,400 surrogate datasets and computed histograms of the model selections for the appropriate Fourier modes based on the logarithmic evidence and the coupling function powers $\int_{0}^{2\pi }{\left|\Gamma (\psi )\right|}^{2}d\psi$. If the coupling function did indeed exist, these coupling function properties would be different between the original and surrogate data. All the surrogate data histograms showed that the M = 0 and $\int_{0}^{2\pi }{\left|\Gamma (\psi )\right|}^{2}d\psi$ = 0 cases were the high frequent. The original coupling functions from speech sound to EEG activity, Γ_*θ*,*s*_ and Γ_*θ*,*p*_, could not explain the surrogate data histograms. In contrast, the coupling functions from EEG activity to speech sound, Γ_*s*,*θ*_ and Γ_*p*,*θ*_, were similar to the surrogate data results.(a) Histograms of the M values which were selected based on logarithmic evidence for the coupling functions Γ_*θ*,*s*_. The blue line represents the model selection histogram for the 1,400 surrogate datasets, while the red line represents the model selection histogram for the 14 participants’ original data. (b) Histogram of all coupling function powers for the surrogate data (including M = 0,1,2,3). The red stars represent the coupling function powers for the original data. (c) Model selection histograms for the coupling functions Γ_*s*,*θ*_. (d) Histogram of powers of the coupling functions Γ_*s*,*θ*_. (e) Model selection histograms for the coupling functions Γ_*θ*,*p*_. (f) Histogram of powers of the coupling functions Γ_*θ*,*p*_. (g) Model selection histograms for the coupling functions Γ_*p*,*θ*_. (h) Histogram of powers of the coupling functions Γ_*p*,*θ*_.(TIF)
