# Supplementary material for: A dynamical systems approach for estimating phase interactions between rhythms of different frequencies from experimental data
Source: PLoS Comput Biol. 2018 Jan 16;14(1):e1005928. doi: 10.1371/journal.pcbi.1005928 (PMC5770039; doi:10.1371/journal.pcbi.1005928)
Supplement: S3 File — (PDF) [file pcbi.1005928.s003.pdf]

S3 File. Hyperparameters of prior distribution and updating rule.

We explain the Bayesian linear regression for estimating phase coupling functions for cross-frequency synchronization in the Materials and Methods section of the main text. Here, we describe how the posterior distribution parameters are computed.

Using Eq. 12 with Eqs. 11 and 13, we can compute the posterior distribution parameters as follows:

$$\boldsymbol{\chi}_i^{\boldsymbol{new}} = \boldsymbol{\Sigma}_i^{\boldsymbol{new}}(\mathbf{F}_i{}^T\mathbf{d}_i + \boldsymbol{\Sigma}_i^{\boldsymbol{old}^{-1}}\boldsymbol{\chi}_i^{\boldsymbol{old}})$$

$$\boldsymbol{\Sigma}_i^{\boldsymbol{new}} = (\boldsymbol{\Sigma}_i^{\boldsymbol{old}^{-1}} + \mathbf{F}_i{}^T\boldsymbol{F}_i)^{-1},$$

$$\alpha_i^{new} = \alpha_i^{old} + \frac{T}{2},$$

$$\beta_i^{new} = \beta_i^{old} + \frac{1}{2}\left(\boldsymbol{d}_i{}^T\boldsymbol{d}_i + \boldsymbol{\chi}_i^{\boldsymbol{old}^T}\boldsymbol{\Sigma}_i^{\boldsymbol{old}^{-1}}\boldsymbol{\chi}_i^{\boldsymbol{old}} - \boldsymbol{\chi}_i^{\boldsymbol{new}^T}\boldsymbol{\Sigma}_i^{\boldsymbol{new}^{-1}}\boldsymbol{\chi}_i^{\boldsymbol{new}}\right).$$

We determine the phase velocity vector and the matrices of the bases of the Fourier series at each time as follows:

$$\mathbf{d}_i = [(\phi_i(t_2) - \phi_i(t_1))/\Delta t \;\; (\phi_i(t_3) - \phi_i(t_2))/\Delta t \;\; (\phi_i(t_{T+1}) - \phi_i(t_T))/\Delta t]^T,$$

$$\boldsymbol{F}_i = \begin{bmatrix} 1 & \boldsymbol{G}_{i,1}(\psi_{i,1}(1)) & \cdots & \boldsymbol{G}_{i,i-1}(\psi_{i,i-1}(1)) & \boldsymbol{G}_{i,i+1}(\psi_{i,i+1}(1)) & \cdots & \boldsymbol{G}_{i,N}(\psi_{i,N}(1)) \\ & & & \vdots & & & \\ 1 & \boldsymbol{G}_{i,1}(\psi_{i,1}(T)) & \cdots & \boldsymbol{G}_{i,i-1}(\psi_{i,i-1}(T)) & \boldsymbol{G}_{i,i+1}(\psi_{i,i+1}(T)) & \cdots & \boldsymbol{G}_{i,N}(\psi_{i,N}(T)) \end{bmatrix},$$

$$\boldsymbol{G}_{ij}(\psi_{ij}) = [\cos\psi_{ij} \;\; \sin\psi_{ij} \;\; \cos 2\psi_{ij} \;\; \sin 2\psi_{ij} \;\; \cdots \;\; \cos \mathrm{M}_{ij}\psi_{ij} \;\; \sin \mathrm{M}_{ij}\psi_{ij}],$$

where, $\psi_{i,1}(t)$ is phase difference $p_i\phi_j(\tau) - p_j\phi_i(\tau)$. Then, we set the hyperparameters of the prior distribution to the following values:

$$\boldsymbol{\chi}_i^{\boldsymbol{old}} = \mathbf{0}, \alpha_i^{old} = \beta_i^{old} = 0, \lambda = 1,$$

$$\boldsymbol{\Sigma}_i^{\boldsymbol{old}} = \mathrm{diag}[\lambda^{-1}, \lambda^{-1}, \lambda^{-1}, 2\lambda^{-1}, 2\lambda^{-1}, \cdots, M_{i1}\lambda^{-1}, M_{i1}\lambda^{-1}, \lambda^{-1}, \lambda^{-1}, 2\lambda^{-1}, 2\lambda^{-1}, \cdots$$
$$\cdots, M_{i2}\lambda^{-1}, M_{i2}\lambda^{-1}, \cdots, \lambda^{-1}, \lambda^{-1}, 2\lambda^{-1}, 2\lambda^{-1}, \cdots, M_{iN}\lambda^{-1}, M_{iN}\lambda^{-1}],$$

where the size of the matrix of $\boldsymbol{\Sigma}_i^{\boldsymbol{old}}$ is $(1 + \sum_{j \neq i} 2M_{ij}) \times (1 + \sum_{j \neq i} 2M_{ij})$ and λ is a precision parameter. This Bayesian method was proposed and explained in a previous study [1].

1. Ota K, Aoyagi T. Direct extraction of phase dynamics from fluctuating rhythmic data based on a Bayesian approach. ArXiv e-prints [Internet]. 2014 May 1, 2014; 1405. Available from: http://adsabs.harvard.edu/abs/2014arXiv1405.4126O.
