# Supplementary figures and images for: A dynamical systems approach for estimating phase interactions between rhythms of different frequencies from experimental data
Source: PLoS Comput Biol. 2018 Jan 16;14(1):e1005928. doi: 10.1371/journal.pcbi.1005928 (PMC5770039; doi:10.1371/journal.pcbi.1005928)

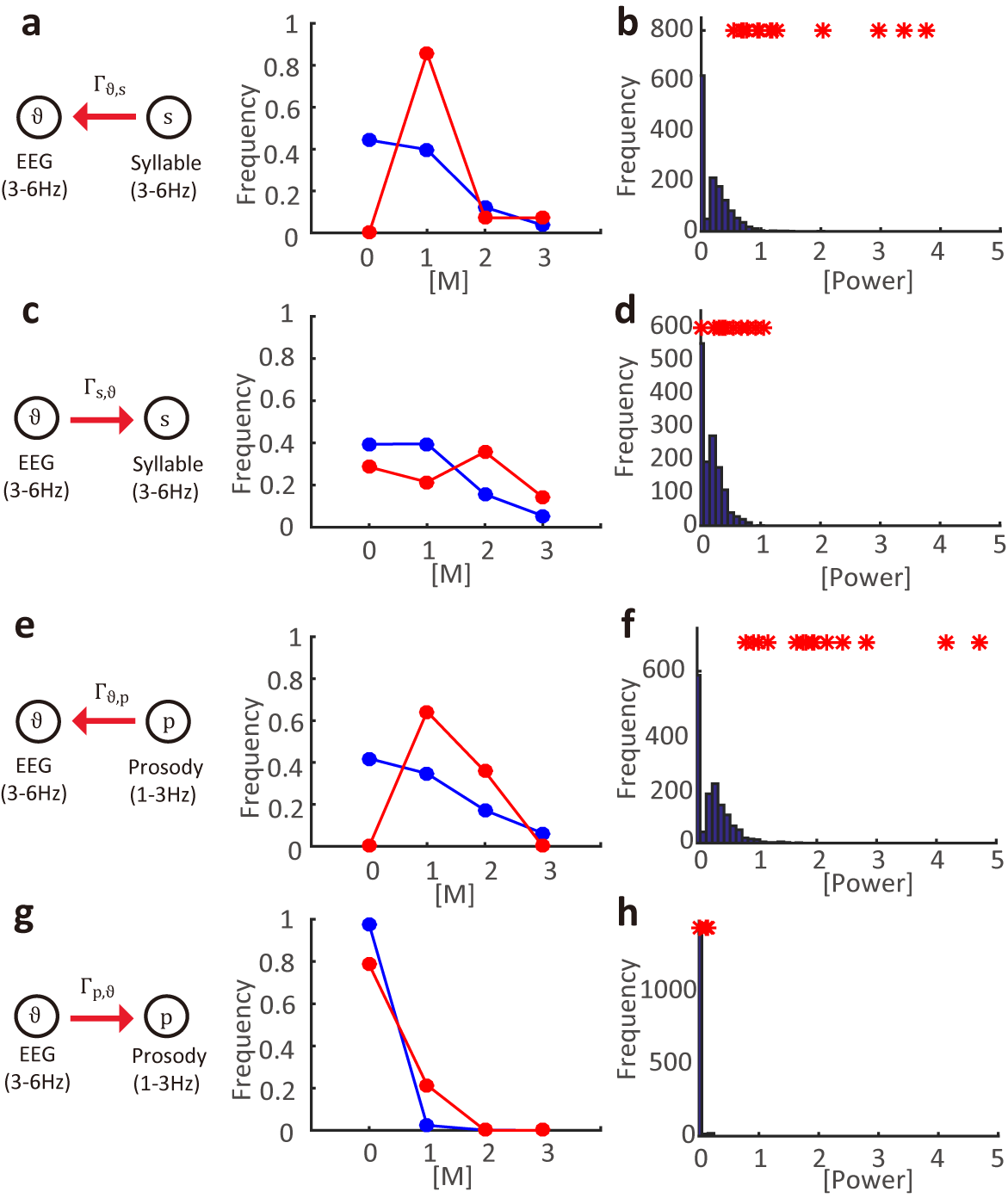

Supplement: S1 Fig — We estimated the coupling functions for surrogate data which have no temporal relationship between the EEGs and speech sounds for comparing the estimated results with the original data. The surrogate data consisted of the original EEG phases and time-shifted speech sound phases. The speech sound phases were randomly time-shifted for each trial so that there would be no temporal relationship between the EEG and speech sound phases. This random shifting process was repeated 100 times for each of the 14 participants. We then estimated the coupling functions using these 1,400 surrogate datasets and computed histograms of the model selections for the appropriate Fourier modes based on the logarithmic evidence and the coupling function powers ∫02π|Γ(ψ)|2dψ. If the coupling function did indeed exist, these coupling function properties would be different between the original and surrogate data. All the surrogate data histograms showed that the M = 0 and ∫02π|Γ(ψ)|2dψ = 0 cases were the high frequent. The original coupling functions from speech sound to EEG activity, Γθ,s and Γθ,p, could not explain the surrogate data histograms. In contrast, the coupling functions from EEG activity to speech sound, Γs,θ and Γp,θ, were similar to the surrogate data results. (a) Histograms of the M values which were selected based on logarithmic evidence for the coupling functions Γθ,s. The blue line represents the model selection histogram for the 1,400 surrogate datasets, while the red line represents the model selection histogram for the 14 participants’ original data. (b) Histogram of all coupling function powers for the surrogate data (including M = 0,1,2,3). The red stars represent the coupling function powers for the original data. (c) Model selection histograms for the coupling functions Γs,θ. (d) Histogram of powers of the coupling functions Γs,θ. (e) Model selection histograms for the coupling functions Γθ,p. (f) Histogram of powers of the coupling functions Γθ,p. (g) Model selectio [file pcbi.1005928.s004.tif]
